# From Fibroadenoma to Phyllodes Tumor: Case Analysis of Borderline and Giant Breast Tumors with Literature Review

**DOI:** 10.15388/Amed.2025.32.1.4

**Published:** 2025-02-18

**Authors:** Justė Kazlauskaitė, Iryna Schmeil

**Affiliations:** 1 Faculty of Medicine, Vilnius University, Vilnius, Lithuania; 2 Department of Gynecology and Obstetrics, Uster Hospital, Uster, Switzerland cwe

**Keywords:** phyllodes tumor, giant phyllodes tumor, filoidinis navikas, didžiulis filoidinis navikas

## Abstract

**Background:**

Phyllodes tumors are highly uncommon fibroepithelial neoplasms of the breast, accounting for less than 1% of all breast tumors. Differential diagnosis between phyllodes tumors and fibroadenomas by using imaging techniques such as ultrasound or mammography, as well as histological methods, can be challenging due to overlapping features. Phyllodes tumors are categorized into benign, borderline, and malignant types, each posing a different risk of recurrence and metastasis. Despite many advances in the imaging and biopsy techniques, diagnosing phyllodes tumors remains challenging. The purpose of this study is to review the existing literature on this topic and describe two cases of misdiagnosed phyllodes tumors.

**Materials and methods:**

A literature review was conducted by using the *Medline* (*PubMed*) database over 10 years. Information concerning the patients was sourced from the Uster Hospital database. After analyzing the cases of women with breast lumps from 2020 to 2023 in the Uster Hospital database, two cases of misdiagnosed phyllodes tumors were identified. These two cases were analyzed retrospectively.

**Results:**

A retrospective study of two cases confirms that phyllodes tumors are a rare phenomenon. A 51-year-old premenopausal woman presented with an 8 × 4 × 5 cm mass, initially diagnosed as a fibroadenoma. The final histopathology after surgical excision revealed a borderline phyllodes tumor with features overlapping those of a fibroadenoma. The second case involved a 59-year-old postmenopausal woman with a rapidly growing mass, which reached 11.9 × 11.3 cm and was initially diagnosed as a fibroadenoma but later confirmed as a borderline malignant phyllodes tumor with focal malignant components. Both cases highlight the limitations of imaging and core biopsy in accurately diagnosing phyllodes tumors and emphasize the need for comprehensive histopathological evaluation. The described clinical cases corresponded to the characteristics of phyllodes tumors indicated in the literature: they appeared in women older than 35 years, were hard to distinguish from fibroadenomas, and required surgical treatment.

**Conclusions:**

Phyllodes tumors are challenging to distinguish from fibroadenomas based on imaging and the initial biopsy results alone. Accurate diagnosis requires thorough histopathological examination following surgical excision. A multidisciplinary approach is essential for optimal management. Our cases show the complexity of phyllodes tumor diagnosis and the importance of considering phyllodes tumors in the differential diagnosis of breast masses, especially when clinical and imaging findings suggest a more aggressive pathology.

## Abstract (it)

**Įvadas:**

Filoidiniai navikai yra labai retai pasitaikantys krūties fibroepiteliniai navikai. Šie dariniai sudaro mažiau nei 1 proc. visų krūties navikų. Filoidinio naviko ir fibroadenomos diferencinė diagnostika naudojant vaizdinimo, pavyzdžiui, ultragarso ar mamografijos, bei histologinius metodus, gali būti sudėtinga dėl panašių šių navikų požymių. Filoidiniai navikai skirstomi į gerybinius, ribinius ir piktybinius tipus, kiek-vienas iš šių tipų kelia skirtingą atsinaujinimo ir metastazių riziką. Nepaisant vaizdinimo ir biopsijos metodų pažangos, diagnozuoti filoidinį naviką vis dar sudėtinga. Šio tyrimo tikslas – apžvelgti esamą literatūrą šia tema ir aprašyti du klaidingai diagnozuotų filoidinių navikų atvejus.

**Medžiaga ir metodai:**

Buvo atlikta 10 metų literatūros apžvalga *Medline (PubMed)* duomenų bazėje. Informacija apie pacientes gauta iš Usterio ligoninės duomenų bazės. Išanalizavus 2020–2023 m. Usterio (Šveicarija) ligoninės duomenų bazėje esamus moterų, kurioms buvo nustatyti krūties navikai, atvejus, išaiškinti du klai-dingai diagnozuotų filoidinių navikų atvejai. Šie du atvejai buvo analizuojami retrospektyviai.

**Rezultatai:**

Retrospektyvus dviejų atvejų tyrimas patvirtina faktą, kad filoidiniai navikai yra retas reiškinys. 51 metų moteris prieš menopauzę kreipėsi dėl 8×4×5 cm darinio, iš pradžių diagnozuoto kaip fibroadenoma. Galutinė histopatologinė diagnozė po chirurginio gydymo atskleidė esant ribinį filoidinį naviką, kurio požymiai sutapo su fibroadenomos požymiais. Antrasis atvejis – 59 metų moteris po menopauzės, kuriai buvo nustatytas sparčiai augantis 11,9×11,3 cm darinys, jis iš pradžių diagnozuotas kaip fibroadenoma, bet vėliau patvirtintas ribinio piktybiškumo filoidinis navikas su židininiais piktybiniais komponentais. Abiem atvejais išryškėja vaizdinimo ir biopsijos trūkumai tiksliai diagnozuojant filoidinį naviką ir pabrėžiama būtinybė atlikti išsamų histopatologinį vertinimą. Aprašyti klinikiniai atvejai atitiko literatūroje nurodytus filoidinio naviko požymius: atsirado vyresnėms nei 35 metų moterims, buvo sunkiai atskiriami nuo fibroadenomų, pacientėms buvo reikalingas chirurginis gydymas.

**Išvada:**

Filoidinius navikus sunku atskirti nuo fibroadenomų remiantis vien tik vaizdinimo ir pirminės biopsijos rezultatais. Tiksliai diagnozei nustatyti reikia atlikti išsamų histopatologinį tyrimą po chirurginio gydymo. Optimaliam gydymui būtinas daugiadisciplininis požiūris. Mūsų atvejai rodo, kad filoidinio naviko diagnozė yra sudėtinga, svarbu atsižvelgti į filoidinį naviką diferencinėje krūties darinių diagnostikoje, ypač kai klinikiniai ir vaizdinimo duomenys rodo agresyvesnę patologiją.

## Introduction

*Phyllodes tumors* (PT) and *fibroadenomas* (FA) are fibroepithelial neoplasm tumors of the breast, and, due to some of their similarities, diagnostic problems tend to occur [1]. Classifying these tumors remains challenging in core biopsy and excision specimens [2]. Differentiating between PT and fibroadenomas (FA) by using *ultrasound* (US) and mammography is difficult [3]. The most common problem at the benign end of the spectrum is the differentiation of a benign PT from a cellular FA, whereas, at the opposite end of the histological spectrum, malignant PTs must be differentiated from metaplastic carcinomas and sarcomas [2]. While FA is a common benign tumor usually found in younger women, slow-growing, and without risk of malignancy, PT is rarer, as it accounts for less than 1% of all breast tumors, and is usually found in middle-aged women, is often fast-growing and prone to recurrence or metastasize [4,5]. Histologically, PT can be divided into benign (60%), borderline (20%), and malignant (20%) [3]. Typically, FA and PT are painless, firm, and mobile breast lumps, varying from 1 cm to over 10 cm (median size 4 to 7 cm) [6]. Tumors larger than 10 centimeters are called giant tumors; this occurs in 20% of PT cases. FA and PT are often difficult to distinguish when they first appear because they are both well-circumscribed, lobed masses, and the core needle biopsy has a sensitivity of only 75% [1]. It is important to differentiate PT from FA, since PT is more aggressive and requires more radical treatment than FA [2]. This study aims to review the existing literature on this topic and describe two cases of PT, with particular attention to the common diagnostic and practical problems, and to highlight the importance of applying new research methods to avoid misdiagnoses.

## Materials and Methods

### Study design and data source

**Fig. 1:**
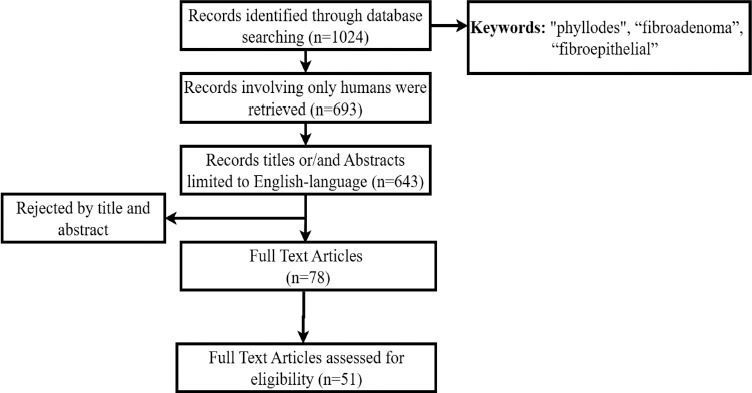
Literature review flowchart

A literature review was conducted in the *Medline* (*PubMed*) database over 10 years (see Fig. 1). A detailed search, including the keywords ‘phyllodes’, ‘fibroadenoma’ and ‘fibroepithelial’ revealed 1024 records. 693 articles involving only humans were retrieved. The study was limited to English-language publications (643 in total), revealing 51 articles.

### Identification of cases

A retrospective review of 2 cases confirms that PT is a rare phenomenon. The clinical cases described fit the characteristics found in the literature: they mostly occur in women over 35 years old, are difficult to distinguish from FA, and the patients require surgical treatment. Information concerning the patients was obtained from the Uster Hospital (Switzerland) database. After analyzing the database from the years 2020 to 2023, two cases of PT were found. Due to several key factors, these cases were selected to demonstrate the diagnostic complexity between PT and FA. In both cases, large breast masses were found, and their imaging and histopathological findings initially suggested FA, which is a far more common diagnosis than PT. However, histology after excision revealed PTs. These cases highlight that current clinical, imaging, and histopathological assessment methods still present difficulties in terms of distinguishing between PT and FA, and therefore the integration of new techniques, such as *artificial intelligence* (AI) integration into imaging analysis, new molecular markers, and genetic testing could help improve the diagnostic accuracy.

## Case Reports

### Case 1

**Fig. 2:**
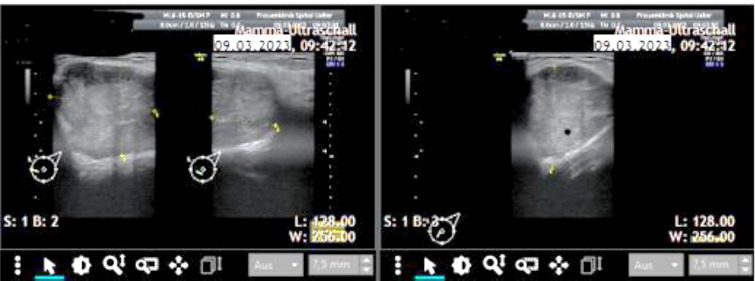
Ultrasonography results. Case 1

**Fig. 3:**
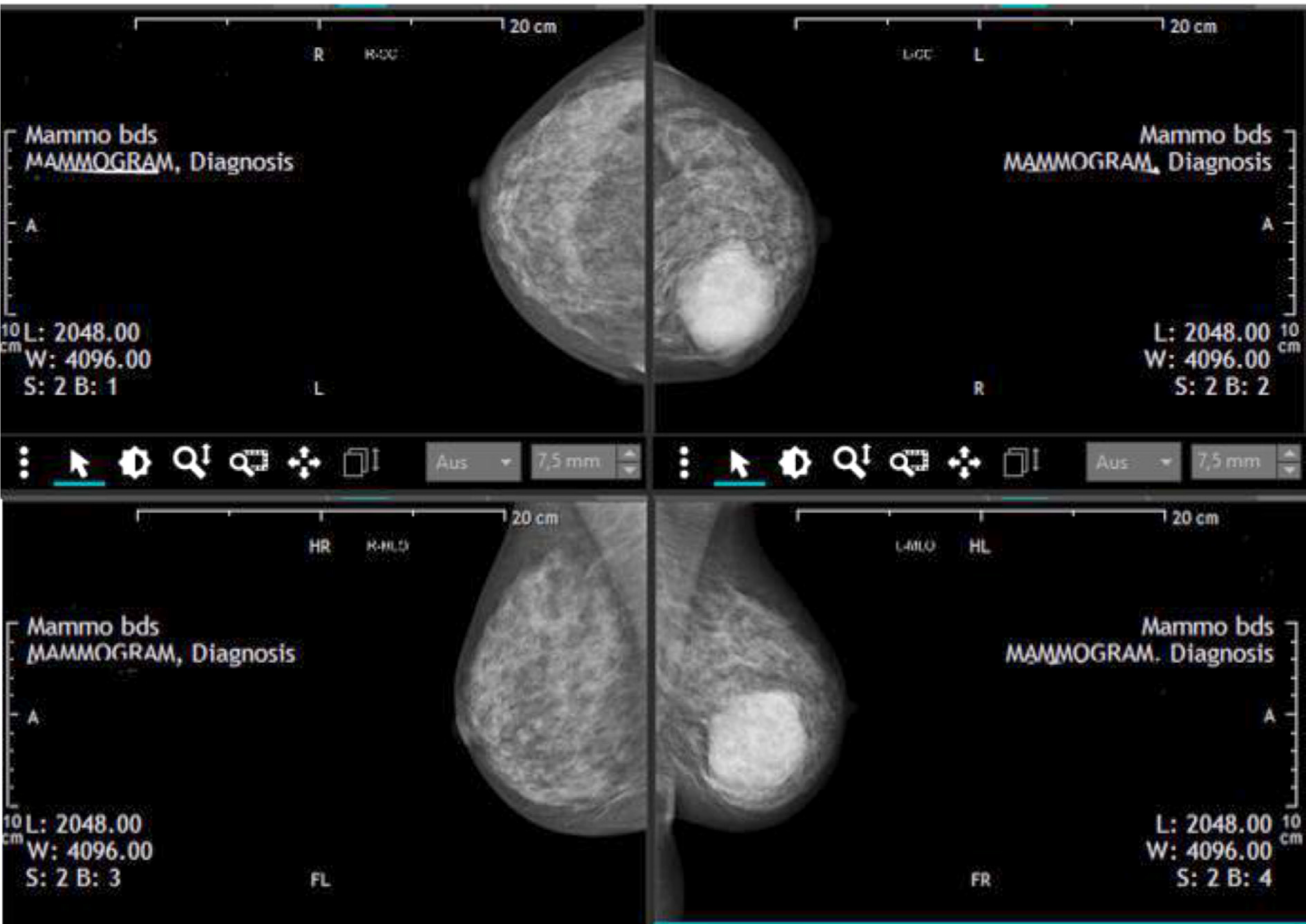
Ultrasonography results. Case 1

**Fig. 4:**
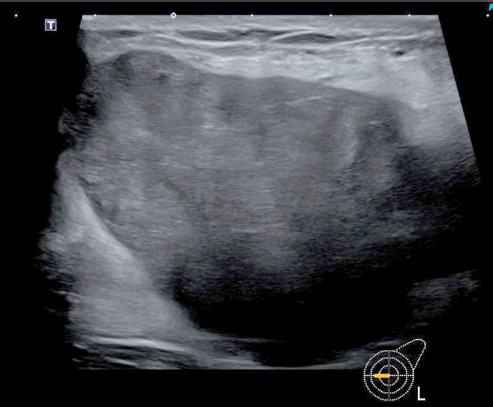
Mammography results. Case 1

A 51-year-old pre-menopausal patient presented with palpable findings which had progressed in size in a few months. The patient did not have any other symptoms apart from the palpable mass. In the clinical examination, an approximately 8 cm large, well-mobile mass could be felt in the left breast in the upper and lower inner quadrant area. Both breasts were asymmetrical in favor of a lower side (due to a large tumor in the area of the left breast); no nipple secretion on either side could be seen. Breast sonography (Fig. 2, Fig. 3) on the left side revealed a round focal mass from 7 to 11 o’clock, the size of the lump was 8 × 4 × 5 cm. Mammography (Fig. 4) revealed dense fibro-glandular tissue in both breasts, which limited the sensitivity for detecting small focal abnormalities. An irregular hyperdense focal finding measuring 64 x 54 mm was observed on the left breast between the 9 and 11 o’clock positions, approximately 3 cm from the nipple. No suspicious calcifications were noted. The axillae appeared clear. A punch biopsy was then performed, and the histological results showed FA. Histological clarification was carried out, which revealed FA with no evidence of malignancy. The operation was planned for a giant benign tumor. The final histological processing of the specimen revealed borderline PT. The morphology went beyond a benign PT – however, the present histological criteria for a malignant PT were not fulfilled. The histopathological diagnosis after operation was as follows: borderline PT with prominent FA-like areas, as well as focal *lobular neoplasia* (LN, ALH). Macroscopically: numerous tissue fragments of 84 g in the fixed state. Immunohistochemistry: Ki 67, p53. P63 LN: e-cadherin (neg), p120 (cytoplasmatic).

### Case 2

**Fig. 5:**
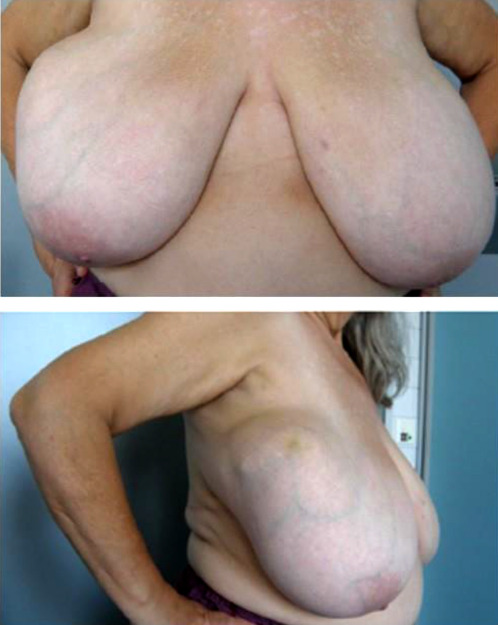
Breast tumor in the right breast. Case 2

**Fig. 6:**
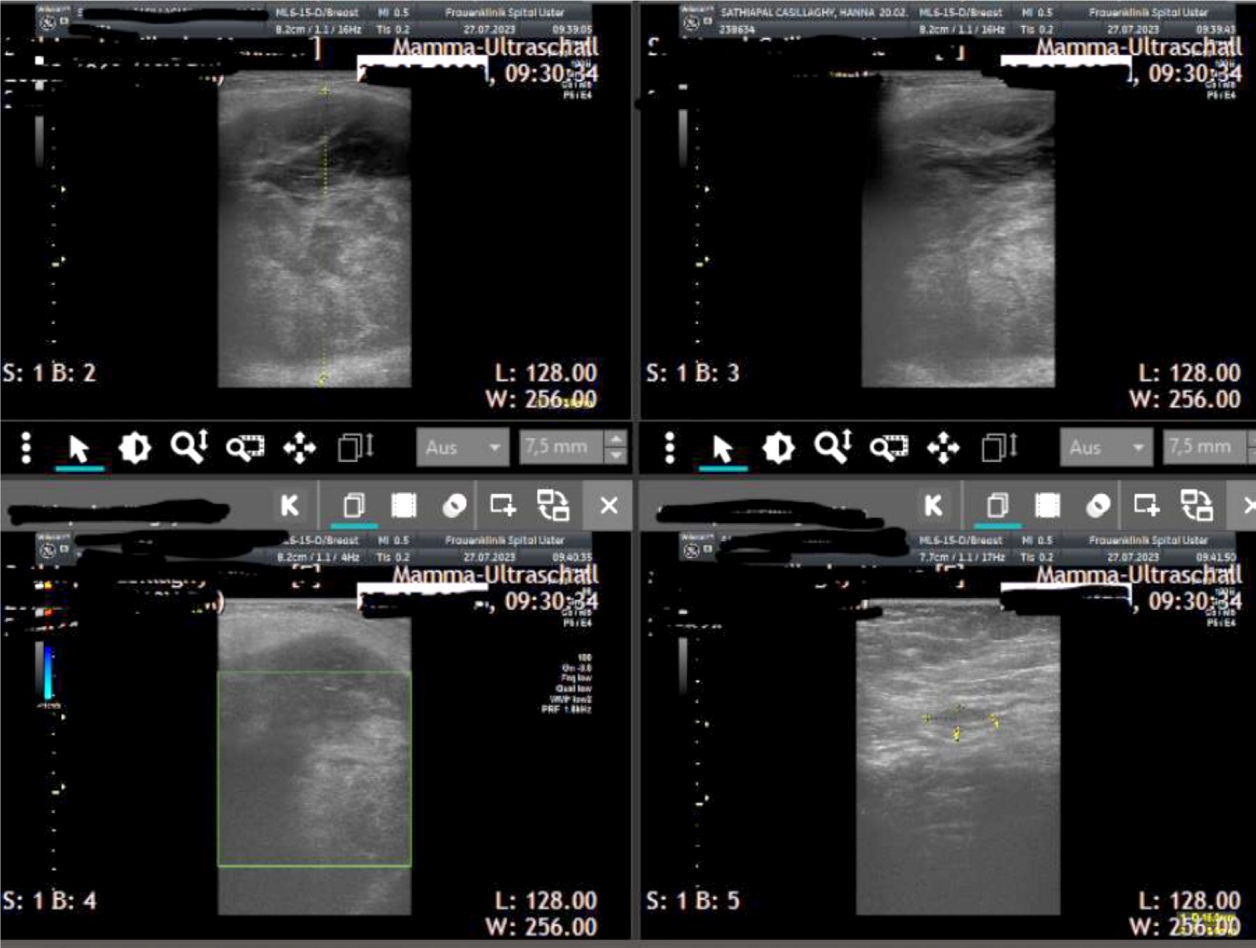
Ultrasonography results. Case 2

**Fig. 7:**
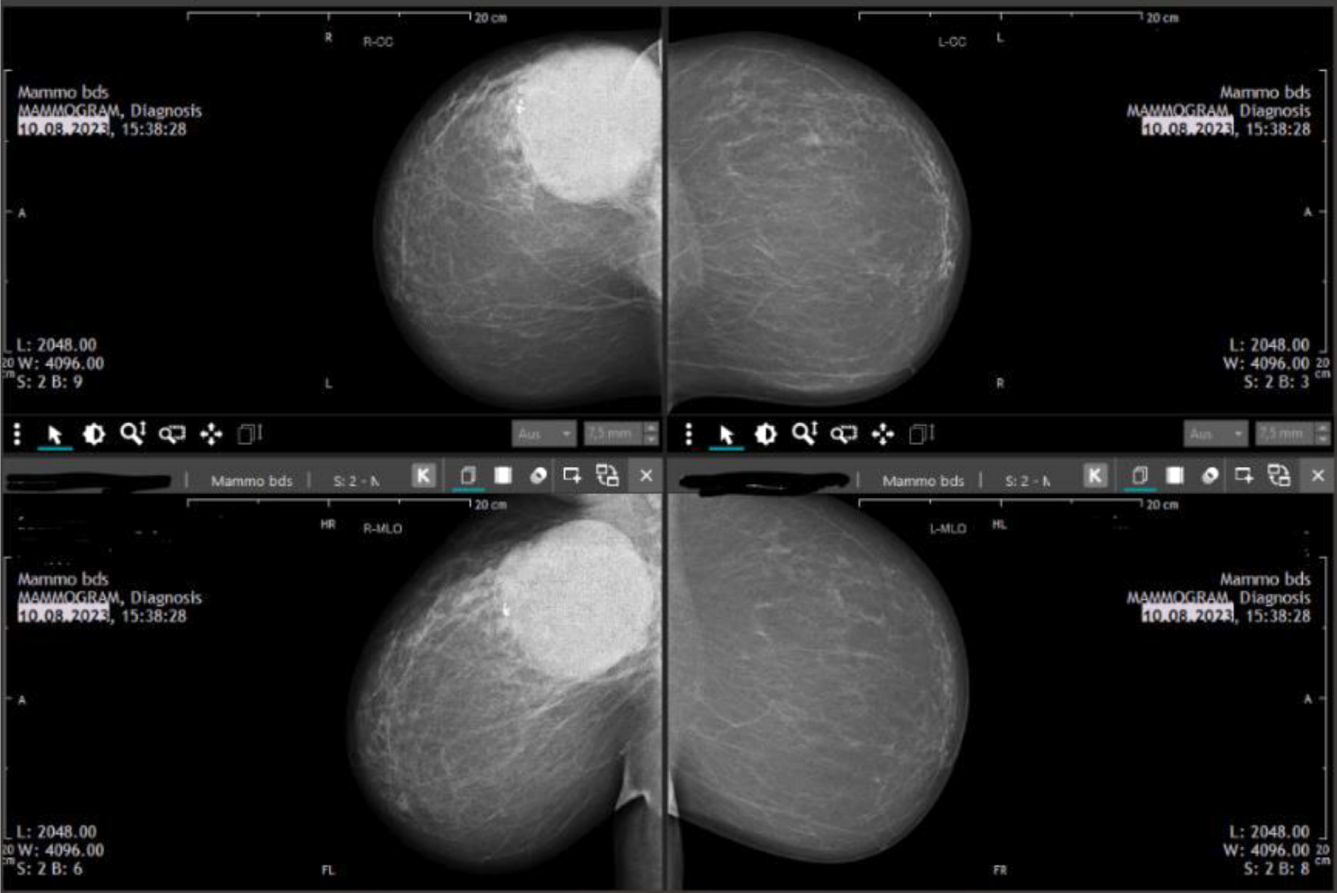
Mammography results. Case 2

A 59-year-old postmenopausal patient was diagnosed with a breast tumor located in the upper right quadrant. Punch biopsy initially identified a lesion as B2 (FA). However, imaging (ultrasound *breast imaging-reporting and data system* (BI-RADS) score 5, and mammography BI-RADS score 4) classified the findings as malignant. Based on these findings, surgical removal of the mammary tumor was recommended. The patient was hesitant to undergo surgery, and the mass was reported to have disappeared after the biopsy. However, 11 months later, the patient returned as the lump reappeared and grew significantly larger during self-examination. The patient’s family history included her mother, who had melanoma at age 80, and her aunt, who died from an unknown cancer at age 70. During clinical examination, a lump larger than 10 cm in size could be palpated in the upper right breast, the tumor was large and visible (Fig. 5), and filled the entire upper outer quadrant. The nipple-areola complex on both sides appeared normal upon inspection. In sonography (Fig. 6), a 10 × 8 cm, a smooth-edged tumor can be seen in the upper right breast. The tumor was multilocular and solid with a displacing effect and partial necrosis, and the overall findings corresponded to at least BIRADS 4. Axilla on the right was without pathologically conspicuous lymph nodes. Only one prominent lymph node with the preserved structure could be observed. Mammography (Fig. 7) was then performed, which showed an unremarkable appearance of the cutis, subcutis and nipple region. Partially covered, otherwise smoothly circumscribed hyperdense mass was observed with a maximum diameter of 11.9 × 11.3 cm on the right side at 11:00 o’clock with marginal calcification. Additionally, global asymmetry favors the right side in the upper outer quadrants. Individual benign calcifications were noted. No suspicious calcifications were found. Barely captured axillary lymph nodes were observed; as far as shown, they were inconspicuous. The punch biopsy results revealed FA with areas of necrosis. A breast surgery for benign changes was performed, and histology results came. Resectate was a PT of the breast measuring up to 11 cm with borderline malignancy and a focal malignant component.

The findings of these two cases are summarized in Table 1.

**Table 1:** Two cases where PT was found

	Case 1	Case 2
*Gender*	Female	Female
*Age*	51 years (pre-menopausal)	59 years (post-menopausal)
*Initial Symptoms*	Palpable mass, no other symptoms	Palpable mass, initially no other symptoms, significant growth noted after 11 months
*Clinical Findings*	8 cm well-mobile mass in the left breast, asymmetry noted	Palpable lump larger than 10 cm in upper right breast, normal nipple-areola complex
*Imaging*	Ultrasound: 8 × 4 × 5 cm round focal mass	*Ultrasound:* 10 × 8 cm smooth-edged tumor, multiloculated and solid, partial necrosis, BI-RADS score 4
	*Mammography*: dense fibro-glandular tissue; hyperdense focal finding measuring 64 × 54 mm, no suspicious calcifications	*Mammography*: 11.9 × 11.3 cm hyperdense mass with marginal calcification, global asymmetry favoring the right side
*Nipple discharge*	No	No
*Histopathological Findings*	*Core biopsy*: FA. *Final diagnosis*: borderline PT with prominent FA-like areas and lobular neoplasia (LN, ALH)	*Core biopsy*: FA with necrosis. *Final diagnosis*: PT with borderline malignancy and focal malignant component
*Immunohistochemistry*	Ki 67, p53, P63, E-cadherin (negative), p120 (cytoplasmic)	Not specified
*Surgical Intervention*	Planned for a giant benign tumor, final histology showed borderline PT	Breast surgery was performed for benign changes, histology revealed borderline malignancy
*Family History*	Not provided	Mother with melanoma at age 80; aunt died from unknown cancer at age 70

## Discussion

FA and PT are fibroepithelial neoplasms consisting of proliferative epithelial and stromal components. FA accounts for 20–50% of breast biopsies, whereas PT accounts for <1% of all breast tumors [4,7]. These tumors are most commonly found incidentally during US examination or by self-palpation as a breast lump [4]. According to the data of the *World Health Organization* (WHO), PT is divided into malignant, borderline, and benign categories based on histological characteristics: the nature of the tumor boundaries; the degree of atypia; the number of mitoses; the degree of the stromal cell layer and stromal overgrowth [2]. The presumed clinical importance of PT assessment is to predict the clinical behavior. Malignant PT belongs to the very high-risk group of metastases, borderline tumors have a low risk of metastases, but, like benign tumors, they often recur locally [8]. As much as 80% of all PTs are benign and borderline. Very often they are misdiagnosed as FAs [9]. According to Karim et al., it is a common procedure to perform a triple examination of the breast lump, and the accuracy of the assessment is very important in the detection of breast cancer. Physical examination, imaging (ultrasound and mammography), and core needle biopsy are more accurate when used together than when used alone [10]. Sars et al. emphasize that the traditional triple assessment approach, which includes breast examination, imaging, and biopsy, often lacks the diagnostic accuracy needed for detecting phyllodes tumors. Given this gap, we have developed a practical guidance table aimed at assisting in more accurately differentiating FA from PT, as evidence-based guidelines for this are currently insufficient [11]. Table 2 outlines key imaging, histology, growth patterns and molecular markers characteristics that aid in differentiating PT from FA.

**Table 2:** Key imaging, histology, growth patterns and molecular markers characteristics distinguishing PT from FA

Differentiating aspect	Phyllodes tumors	Fibroadenomas
*Ultrasound*	Large (> 3 cm); lobulated masses; irregular or ill-defined margins; heterogeneous internal echoes with cystic spaces; linear fluid-filled clefts; necrosis (borderline/malignant PT), vascularity (malignant PT) [12,13]	Well-circumscribed; round or oval masses; smooth margins; homogeneous internal echo pattern; lacks cystic spaces; lacks necrosis [12,13]
*Mammography*	Large; well-defined masses; lobulated or partially circumscribed borders; high-density stromal tissue; cystic areas; calcifications [14,15]	Oval masses; well-defined borders; calcifications (older FA) [14,15]
*MRI*	Heterogeneous; rapid initial contrast uptake, delayed washout; irregular internal architecture; cystic degeneration; necrosis (malignant PT) [9]	Homogeneous; gradual contrast uptake; well-defined margins; lacks cystic changes or architectural distortion [9]
*Core needle biopsy*	Stromal overgrowth; cellular atypia; increased mitotic activity (malignant cases) [16]	Uniform stromal and epithelial components; minimal atypia; low mitotic activity [16]
*Growth pattern*	Rapid growth; recur if not fully excised; higher likelihood of becoming malignant [4,5]	Slow growth; rarely recurs; generally benign [4,5]
*Histology*	Stromal overgrowth; atypia; increased mitotic activity (malignant PT) [16]	Uniform cellularity; low mitotic activity [16]
*Molecular markers*	Higher Ki-67 and p53 expression (borderline or malignant PT) [17]	Low Ki-67; rare p53 mutations [17]
*Follow-up and recurrence*	Follow-ups are required due to recurrence risk [18]	Minimal follow-up is needed; low risk of recurrence[18]
*Surgical excision*	Excision with clear margin (>1cm) [18]	Monitoring or excision (large FA) [18]

### Radiological assessment

When comparing PTs with FAs, PTs are significantly more likely to be >3 cm, irregular/lobulated in shape, have micro-lobulated indistinct margins [9], a heterogeneous internal echo pattern, absence of microcalcifications, mildly hypoechoic internal echoes [19], hypervascularity, and a BI-RADS score of ≥4 [9,20,21]. Factors such as density, calcification, features of breast parenchyma surrounding mass, or BI-RADS were not significant descriptors to differentiate between the PT grade or between FA and PT [13,17,20-21]. Characteristic sonographic findings include intra-tumoral cystic spaces and linear fluid-filled clefts. Imaging findings of PT may imitate papillary lesions, FA and circumscribed cancers. On mammography, PTs are usually ovoid or lobulated, generally well-circumscribed masses. The US shows solid circumscribed ovoid or lobulated masses or complex cystic masses [12]. Mammography and US methods have limitations in differentiating between PT and benign lesions. They cannot specify its histological grade. Regardless of the histological grade, the clinical course is unpredictable and faces difficulties in terms of including the development of local recurrence, distant metastasis, and the survival rate [13]. US elastography may differentiate FA and PT based on the propensity of stiffness of the lesions (the strain ratio). Li et al. reported a spectrum of the strain ratio (FA < benign PT < malignant PT) [23]. Borderline/malignant PT shows higher stiffness by shear wave elastography than benign lesions [21]. Texture analysis shows promise of distinguishing between simple FA, complex FA, and benign PT [24]. Breast *magnetic resonance imaging* (MRI) is usually offered for selected cases to document the extent of the disease and check the resectability of the tumor.

### Importance of core needle biopsy and excision in the diagnosis of fibroepithelial tumors

Pathological examination of PT remains the most important part of the diagnosis. A core biopsy or excisional biopsy is used for diagnosis [6]. An increased number of stromal cells in PT and FA is an important indicator of both of these tumors; therefore, the differential diagnosis of PT and FA remains challenging [8]. PTs are biphasic and have a leaf-like structure with abundant cellular stroma, distinguishing them from stromal sarcomas, spindle cell metaplastic carcinomas, and FAs. Their distinction is important because their treatment and prognosis are different. In FA, the leaf-like pattern characteristic of PT is usually not seen [25]. In a small sample, PT might have regions that are identical to FA, which could be deceptive. Stromal cellularity/heterogeneity and condensation, atypia, mitoses, and fragmentation are some of the morphologic characteristics that have been utilized to diagnose PT or FA [16]. According to Tariq et al., the diagnostic accuracy of the core needle biopsy diagnosis is about 90.4%. On the core needle biopsy, juvenile FA and cellular FA variations are particularly challenging to distinguish from benign PT. All tumors larger than 5 cm should be strongly considered for PT diagnosis. Making the right diagnosis may be aided by increasing the number of cores and correlation with the radiological and clinical results [26]. According to Zhou et al., there are considerable variations in the accurate diagnosis rates of the core needle biopsy for borderline PT, but most are below 50% [27]. According to Li et al., regarding recent thorough analysis of histological fibroepithelial tumor features on core needle biopsy, a constellation of several histological features should be used to make the diagnosis because there is no single diagnostic histologic feature that can distinguish between these two entities [7]. Furthermore, lesions whose core needle biopsy results conflict with clinical or radiologic findings should be considered for surgical removal. The excisional specimen provides the best view of the lesion’s margins, growth patterns, and architecture [26]. Several studies have shown the need for biomarkers, especially Ki-67, in addition to the histologic features of these lesions for a more accurate preoperative evaluation. According to studies, a low Ki-67 labeling index of <1–2% was indicative of benign PT [7,17].

### Molecular markers

The immunohistochemical pattern is characterized by the expression of p53, Ki-67, CD117, EGFR, p16 and the *vascular endothelial growth factor* (VEGF), which promote angiogenesis and tumor progression, especially in malignant variants [3,28]. Histological criteria remain reliable in classifying benign fibroepithelial tumors, but a genetic study of MED12, TERT promoter, should be performed to better determine the genomic structure of these tumors [29,30]. Table 3 summarizes the main molecular markers reported in the literature to determine PT’s aggressive and malignant potential compared to the usually benign nature of FA.

**Table 3:** Molecular markers and immunohistochemistry characteristics of PT and FA

Molecular marker	Phyllodes tumors	Fibroadenomas
*TERT promoter mutations*	Found in some aggressive PTs, associated with telomere maintenance and tumor progression [29]	Generally lacks these mutations [29]
*MDM2 and CDK4 amplification*	Amplified in malignant PT, associated with cell cycle dysregulation and aggressive growth [31]	Less commonly associated with FA [31]
*MED12 mutation*	Less common and primarily found in benign forms (lower prevalence in borderline or malignant PT) [31]	Higher mutations. Associated with benign behavior [31]
*EGFR (epidermal growth factor receptor)*	Frequently expressed, particularly in malignant PT; may indicate aggressiveness and a potential therapy target [7,17]	Less commonly expressed, rarely significant for diagnosis [7,17]
*RB1 mutation*	Found in malignant PTs [7,29]	Are not typically characteristic of FA [7,29]
*Immunohistochemistry*		
*p53*	A higher frequency of p53 mutations is associated with loss of cell cycle regulation and tumor growth. No p53 mutations in benign PTs [17]	Rare p53 mutations are generally benign and stable [17]
*Ki-67 Proliferation Index*	High Ki-67 (>10%) in malignant PTs, increased cell proliferation and rapid growth [7,17]	Low Ki-67 (<5%); slower cell proliferation; benign behavior [7,17]
*CD34 (Endothelial Cell Marker)*	Frequently positive (malignant PT), indicates vascular endothelial cells and new blood vessel formation [7]	Low expression [7]
*CD117 (c-KIT)*	Can be positive in malignant PTs, associated with an aggressive phenotype [7,17]	Negative, indicates slower growth and stability [7,17]

The local recurrence risk for benign PT varies from 5% to 30%, whereas, for borderline/malignant subtypes, the risk is up to 65%. To minimize this risk, the standard treatment is a wide surgical resection (without the removal of axillary lymph nodes), defined as a negative surgical margin greater than or equal to 1 cm [18]. Side effects are usually rare for all forms of PT when they are completely locally excised [6]. Ulceration of the skin or chest wall invasion may occur in patients with giant PT. Axillary metastases are uncommon; therefore, axillary lymph node staging is usually not necessary [13]. Other changes, like nipple retraction or nipple discharge, are common [6]. Macroscopically, they are well-circumscribed, firm tumors on a gray and mucoid, homogeneous, or cystic surface. In large tumors, areas of hemorrhage and necrosis may be present [13].

The malignant/borderline tumors significantly differed from benign PTs based on a larger size (4–7 cm), an irregular shape, complex cystic echogenicity, presence of clefts, and increased likelihood for a higher BI-RADS score (>3) [8,18,21,22,32]. However, some studies found no association of the size or shape and the presence of cystic spaces with a PT grade [20]. Circumscribed margins, echogenicity, boundaries (an abrupt, echogenic halo), posterior acoustic features, the ratio of length to the anteroposterior diameter, or the mass vascularity were not significant distinguishing features of benign, borderline, and malignant PTs [8,18,20].

Patients with a high probability of a malignant PT with an aggressive clinical course should be investigated. About 22% of malignant PTs have distant metastases. Usually, bones, pancreas due to hematogenous, and lungs are the most common places for the spread of metastases. PT might metastasize to the ovary [33], ileal [34], pelvic area [35], lymph node, and distantly [36]. The degree of atypia in the stroma of a malignant PT is the best predictor of the tumor’s metastatic potential and general behavior [37]. Regardless of other histological signs, the tumor is classified as malignant if a malignant heterologous element is detected [38]. Normal and underweight patients, as well as those with larger tumor sizes, were more prone to local recurrence [39].

### Diagnostic challenges

In this article, we presented two borderline tumors, one of them with a malignant element, that mimicked FA. Although mammography and ultrasound were highly helpful in determining the size of the tumors, the other radiological characteristics of the lesions were quite similar (round focal mass, a smooth-edged tumor, multiloculated and solid, partial necrosis). Mammography showed a defined, hyperdense mass of both. Marginal calcifications, seen on ultrasound in the second case, are suggestive of a PT but are only present in a small proportion of lesions. The histological overlap between PT and FA, combined with the limited specimen size, poses a significant diagnostic challenge. Many histologic parameters have been used to define FA and benign PT, but no well-defined cut points have been established, and thus there is a degree of subjectivity in this diagnosis. In our cases, core biopsies were performed, and the specimens were sent for histological evaluation. The histological results indicated fibroepithelial tumors, specifically, FA with and without necrosis. Only after excision, was the final diagnosis and histology made, which revealed borderline PT. For the first case, immunohistochemistry was performed using the markers Ki-67, p53, p63, E-cadherin, and the results were negative. As discussed, PTs can have FA-like areas, which can be misleading in a limited sampling [16]. According to Lin et al.’s study in core biopsy specimens, Ki-67 may be a helpful marker for differentiating benign from borderline PTs, while p16 and pRb may be a helpful combination of markers for differentiating FA from benign PTs [40]. Shubham et al. examined immunohistochemical markers such as Ki-67, p53, and CD10 to aid in the diagnosis and subtyping of PT. Ki-67 and p53 showed significant differences in expression between malignant, borderline, and benign PTs, correlating with the tumor grade. However, CD10 did not correlate with the tumor grades. No single marker reliably distinguishes benign from borderline PTs [41]. Yang et al. found that the Ki-67 index is a useful marker, but has some practical problems. The main issues include no clear standard for grade categorization, along with difficulties in measuring Ki-67 accurately. While Ki-67 is a useful marker, other markers like CD117 and p53 may help differentiate PTs further, but they are not always reliable [17]. In the first case which we presented, the tumor mimicked the appearance of FA on imaging. PT presentation, diagnosis, and management can be quite varied, leading to several interesting and unique case reports. A retrospective analysis was conducted by Tummidi et al. on 70 patients who received histopathologic follow-up for FA or PT, evaluating cytologic criteria in the process. During the study, it was found that the classification is challenging; it is difficult to distinguish PT from FA in benign cases, and there were problems in classifying the three recognized classes of PT. A few benign fibroepithelial tumors do not histologically fit into either FA or PT categories [42]. Our findings align with Zhang et al.’s perspective that the histopathological, immunophenotypic, and proteomic characteristics of low-grade PT and FA show more similarities than differences [43].

In the second case, we presented a giant borderline PT (11.9 ×11.3 cm) with local recurrence. In our case, the giant PT caused breast deformity, although this is very rare. Several cases of giant PTs (Islam et al., 50 × 50 cm; Yap et al., 25 × 32 × 23 cm; Sbeih et al., 20 × 20 × 25 cm) causing breast deformity, tumor ulceration and bleeding have been reported in the literature [44–46]. Wound closure after the excision of a giant PT remains a major challenge for surgeons [44]. The limited literature on this topic does not help in decision-making. Küçükgüven et al. reported the case of a 38-year-old woman who underwent radical mastectomy for a giant malignant PT measuring 33 × 23.5 × 17 cm and weighing 9.150 kg. The initial biopsy showed it to be a malignant mesenchymal tumor, and the differential diagnosis included PT and carcinosarcoma [47]. Schillebeeckx et al. presented the case of a 57-year-old woman who underwent a needle biopsy of the tumor core, which found a fibroepithelial tumor. The tumor measured 22 × 17 × 17 cm and weighed 4.194 kg. After mastectomy, a microscopic examination confirmed a borderline malignant PT [1]. A case involving a 34-year-old surrogate mother with confirmed PT was presented by Faulds et al. The histological examination revealed a giant tumor, measuring 12 × 10 cm, exhibiting the characteristic features of borderline PT. PT during pregnancy is extremely rare, and it is unclear whether there is a relationship between changes in the hormone levels during pregnancy and the development or growth of PT [48]. A case was presented by Zhang et al. involving a 49-year-old female patient who developed a giant borderline PT (dimensions: 10.5 × 7.0 cm) in her left breast, which recurred four years following a bilateral mastectomy performed for a benign PT. Zhang et al. note that it remains uncertain if radical tumor-negative resection margins are necessary for all subtypes of PT. The recommended wide PT resection with ≥1 cm margins is too large for benign PTs, and a positive resection margin increases the recurrence rate [49].

Based on this literature review and our presented case reviews, we can confirm the opinion that additional clinical trials should be conducted in the future to achieve a standardized multidisciplinary approach to PT of the breast. Contrary to findings in the literature, in our cases, neither radiological imaging, core biopsy, nor immunohistochemical markers helped to differentiate PT from FA. The diagnosis of PT was only achieved after surgical resection. Till now, the best practical tip for a cellular fibroepithelial lesion found on the needle biopsy, which is difficult to classify as either FA or PT tumor, is to completely excise it for accurate classification.

### Future research directions

Due to the complex diagnosis of PT and FA, there is an ongoing effort to improve the diagnostic accuracy by using more accurate, less invasive diagnostic methods. Tan P. H. published a study in which AI was used to distinguish FA from PT on core biopsy based on whole slide images. The AI model’s overall accuracy was 87.5% [30]. Artificial intelligence (AI) integration into imaging analysis may aid in identifying subtle differences in the tumor architecture, potentially improving the diagnostic precision. According to Mansour et al., it may be possible to distinguish between PT and FA found on sono-mammography by using AI-assisted mammograms. To help clinicians decide about a conservative therapy or a surgery, the color hue and the anomaly scoring percentage could be employed as a one-setting technique for specification [50]. Tummidi et al. represent a study which found cytological predictors of PT which could help distinguish it from FA. The characteristics to differentiate between these two entities include stromal traits (frayed irregular boundaries, enhanced stromal cellularity with a predominance of spindle cells), increased background spindle cells, and a preponderance of big folded, opened-up epithelial sheets [42]. Next-generation sequencing shows that benign PTs have more frequent mutations, particularly in RB1, EGFR, and TERT promoter genes, than FAs. These molecular markers may contribute to future differential diagnostics for challenging cases, though histologic criteria stay reliable for most classifications. Further genetic research is needed to refine diagnostic precision [29]. Col3A, or collagen type III alpha 1, is a protein involved in the extracellular matrix formation. In PT, Col3A expression progressively increases from benign to borderline to malignant tumors, with higher levels linked to irregular margins and a high mitotic activity. A unique periductal cuffing pattern of Col3A staining is observed in PTs but not in FA, thereby suggesting that Col3A may be a useful adjunct marker for differentiating FA from PT and for assessing the malignancy potential in PTs [51]. In the future differentiation of FA and PT, the Ki-67 index remains a promising marker for grading PTs. Ki-67’s diagnostic accuracy still needs to be refined. Other markers, like Topoisomerase IIα, anaphase-promoting complex 7, and CD117 also hold potential, while p53 expression shows promise in grading PTs, but not in distinguishing FA from PT. Further research is essential to develop a standardized approach [17].

## Conclusions

Phyllodes tumors and fibroadenomas often exhibit overlapping morphological features, particularly in stromal cellularity, thus complicating accurate diagnosis. Given the limitations of imaging alone in distinguishing phyllodes tumor from fibroadenoma, multidisciplinary evaluation is needed, including histopathology, molecular profiling, and immunohistochemistry. The cases we have presented illustrate the diagnostic challenges posed by PT. Although a range of diagnostic methods exist for distinguishing phyllodes tumors, they often fall short in reliably identifying borderline cases. This limitation arises because biopsy samples may fail to capture the representative areas of the tumor, leading to challenges in achieving an accurate diagnosis; therefore, improved criteria and standardized protocols are needed. Future advancements in molecular markers, cytologic analysis, and AI integration offer promise for enhancing the diagnostic precision and informing tailored management approaches for these complex fibroepithelial lesions.
